# The Regional Hydrologic Extremes Assessment System: A software framework for hydrologic modeling and data assimilation

**DOI:** 10.1371/journal.pone.0176506

**Published:** 2017-05-18

**Authors:** Konstantinos M. Andreadis, Narendra Das, Dimitrios Stampoulis, Amor Ines, Joshua B. Fisher, Stephanie Granger, Jessie Kawata, Eunjin Han, Ali Behrangi

**Affiliations:** 1 Jet Propulsion Laboratory, California Institute of Technology, Pasadena, CA, United States of America; 2 Department of Plant, Soil and Microbial Sciences, Michigan State University, East Lansing, MI, United States of America; 3 Department of Biosystems and Agricultural Engineering, Michigan State University, East Lansing, MI, United States of America; 4 International Research Institute of Climate and Society, Columbia University, Palisades, NY, United States of America; Bristol University/Remote Sensing Solutions Inc., UNITED STATES

## Abstract

The Regional Hydrologic Extremes Assessment System (RHEAS) is a prototype software framework for hydrologic modeling and data assimilation that automates the deployment of water resources nowcasting and forecasting applications. A spatially-enabled database is a key component of the software that can ingest a suite of satellite and model datasets while facilitating the interfacing with Geographic Information System (GIS) applications. The datasets ingested are obtained from numerous space-borne sensors and represent multiple components of the water cycle. The object-oriented design of the software allows for modularity and extensibility, showcased here with the coupling of the core hydrologic model with a crop growth model. RHEAS can exploit multi-threading to scale with increasing number of processors, while the database allows delivery of data products and associated uncertainty through a variety of GIS platforms. A set of three example implementations of RHEAS in the United States and Kenya are described to demonstrate the different features of the system in real-world applications.

## Introduction

Water resources management is a major challenge globally, involving tradeoffs between multiple objectives (e.g., water supply, agriculture, hydropower, ecology) and coordination with a heterogeneous set of stakeholders. Consequently, decision-making in the context of water resources management requires that agencies and practitioners have accurate information on water and energy conditions with as much lead time as possible. Such information is often derived from datasets being offered to end users by data producers, i.e. a producer-driven process [1], rather than users running their own product-generating systems. Despite increased efforts for improved interaction between science organizations (i.e. data producers) and end-users [2], adoption of information for management decisions will be accelerated by direct interaction with and customization of the information-producing system [3]. The aforementioned customization would be rather expensive (in terms of resources) if the developers themselves made modifications, which are likely specific to each end-user. In many cases though, the end-users do not have the technical expertise nor the time or resources to easily implement these beneficial customizations or interact with the information system extensively, thus making the need for a system that can be interacted with in a relatively simple and straightforward fashion more important.

Most likely, a water resources information system would require a model that can simulate the hydrologic response at different spatial and temporal scales. Implementation of such a modeling system is hindered by the dearth of observations that can help calibrate and validate its predictions [4]. In data-poor regions these necessary observation datasets can primarily be obtained from satellite observations, which albeit adds to the difficulty of managing ever-increasing data volumes it also offers the opportunity to better constrain hydrologic models through data assimilation [5].

Here, we present a prototype software framework (the Regional Hydrologic Extremes Assessment System, RHEAS) that automates the ingestion of diverse datasets (both observational and model-based), and the deployment of a hydrologic model incorporating data assimilation and facilitating the coupling with other earth science models. This integrated system is primarily geared for easy implementation and customization requiring relatively little input from end users. Numerous modeling systems that integrate different models and observations have been developed in the past and were used as a stepping stone during the design of RHEAS. The difficulty of discovering, downloading and extracting datasets (either observational or model-based) has been previously recognized [6]. As a result, existing hydrologic software have included capabilities of downloading a predetermined set of datasets (e.g. [7]), while other software have leveraged standardized web services for downloading (e.g. [8]) as well as additionally implementing data discovery options (e.g. [9]).

An important aspect of a hydrologic modeling system is the internal representation of the data (input and output), with a variety of standardized file formats existing (e.g. Network Common Data Form, NetCDF or Hierarchical Data Format, HDF). In addition to files, data can be stored within databases (e.g. PostgreSQL) that can represent spatial and geographic objects resulting in model-independent data management. There is an advantage in using a model-agnostic storage option for the data, since that would facilitate their transferability across models [10] and external visualization and analysis software (e.g. [11]). Although some hydrologic modeling software incorporate GIS components internally (e.g. [12]), RHEAS enables the interfacing with GIS software creating a system that can capture, store, analyze and visualize geospatial data.

Data assimilation methods have been used in hydrologic research and applications to merge heterogeneous observations and models in order to improve model predictions [13]. Such algorithms have been incorporated within hydrologic modeling software, either for specific models (e.g. [14–16]) or as generic frameworks (e.g. [17–20]). RHEAS employs a number of assimilation algorithms by using an object-oriented and modular design, similar to software such as the Land Information System (LIS, [21]). By designing software as modular components and abstracting their functionality, code reuse is maximized and the flexibility on the modeling tools used is facilitated.

A detailed description of the RHEAS software is given in the following section, including its architecture, different components and operating modes. A set of three example applications of the developed software framework are presented in the Application examples section, while the software status and some potential future directions are discussed in the final section of the paper.

## Software description

RHEAS is a modular software framework that has been developed at the NASA Jet Propulsion Laboratory (JPL) aiming at facilitating the deployment of water resources simulations and the assimilation of remote sensing observations. At the core of the system lies a hydrologic model, the Variable Infiltration Capacity model, that can be run both in nowcasting (i.e. estimation of the current time period) and forecasting (i.e. estimation for future time periods) modes. The nowcast simulation periods can be arbitrarily long, while forecast simulations depend on the length of the meteorological forecasts. In particular, seasonal forecasts will range between 1 and 6 months while long-term forecasts (e.g. climate projections) can range from 5 to 100 years. A suite of datasets from multiple sources are utilized by the system to either force or assimilate observations into the hydrologic model. Data assimilation can constrain hydrologic simulations leading to improved model states and/or parameterizations, and is explicitly incorporated within RHEAS.

### System architecture

**Fig 1:**
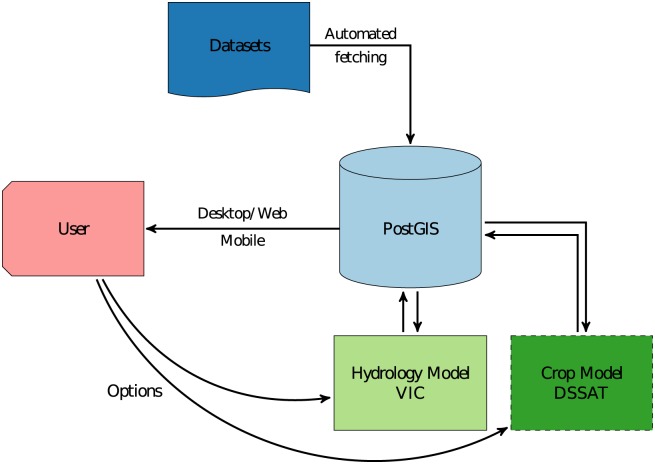
Software architecture. Simplified schematic of the RHEAS software architecture.

Fig 1 shows a schematic of the RHEAS software architecture and its major components. The datasets that are used to perform the model simulations as well as the model outputs are stored within a GIS-enabled relational database (PostGIS), facilitating a model-agnostic dataset format. The latter design choice had several advantages compared to other hydrologic modeling systems: (i) system modularity since the hydrologic model (or any other model that can be added to RHEAS) needs to only interface against the database and not any other model’s internal data formats; (ii) GIS functionality that allows spatial operations, complex queries and analytics on the stored datasets; (iii) ability to serve data through well established technologies (either web, desktop or mobile). The hydrologic model (Variable Infiltration Capacity, VIC) is the primary modeling component within RHEAS, while other models can be coupled (in this case a crop model, DSSAT) extending the system’s applicability. All models contained in RHEAS retrieve their input and store their output in the PostGIS database, which can serve these data to users via different interfaces. The datasets that are not produced by the RHEAS models, including satellite observations and model data that are used to generate inputs or constraints for the models, are automatically fetched from various sources and ingested into the PostGIS database.

**Fig 2:**
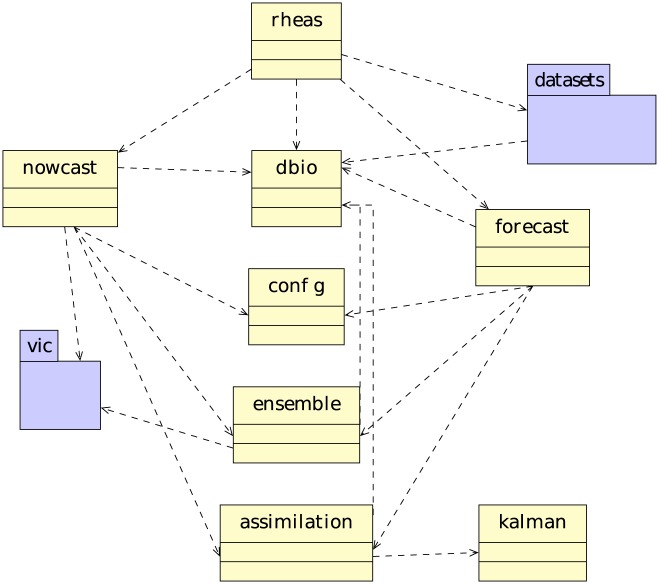
UML component diagram. Unified Modeling Language (UML) diagram describing the components (i.e. modules) within RHEAS.

We designed the RHEAS software following a hybrid approach that combined modular and object-oriented programming. The functionality of the software was broken down into a set of components: (i) configuration, (ii) database operations (I/O and processing), (iii) model simulations, and (iv) data assimilation. Functions associated with each component were encapsulated either within a module or a class if both attributes and methods were needed to describe a component. For example, observations required both attributes (e.g. spatial resolution) and functionality (e.g. retrieve observed variable) and therefore were represented as objects. A simplified UML component diagram is shown in Fig 2 with the main module rheas importing the database (dbio), simulation (nowcast and forecast), and datasets modules. Users can define and perform simulations in RHEAS through a text-based configuration file with parsing functions described in the config module. Most modules need access to the database functionality, hence they import the dbio module. In addition, the simulation modules require the classes defined in the vic package to run the hydrologic model, the ensemble module to perform stochastic simulations, and the assimilation module to assimilate satellite observations.

### Dataset ingestion and storage

A number of earth science datasets are available to be used in RHEAS representing many hydro-meteorological variables (e.g. precipitation), with each of being defined as a class within the datasets package (Fig 2). The PostGIS database, where the RHEAS datasets are stored, is a spatial extension to the widely-used PostgreSQL object-relational database system [22]. The datasets that are organized in tables (i.e. relations) have both a spatial and temporal dimension, with each time snapshot of the data being stored as a row with columns representing the date, a unique identifier key, the actual data and its geographical information. Table 1 shows the structure of each dataset table along with the type of its column. The raster type is represented as a binary blob within PostGIS, and can have multiple bands of georeferenced pixel values.

**Table 1:** Structure of PostGIS tables representing each RHEAS dataset.

rid: integer /∗ unique identifier for each date and raster tile ∗/
fdate: date /∗ date representation ∗/
rast: raster /∗ georeferenced data ∗/

PostgreSQL allows for querying the data by applying various operators (e.g. union, intersection) with arbitrary constraints. PostGIS expands these capabilities to allow processing and analytic functions (e.g. classification, statistics), map algebra, map reprojections, and spatial resampling to coarser or finer resolutions. If the data are represented as rasters, they are split into tiles (by the PostGIS software) with each row containing a specific raster tile for each date to improve performance. Additional optimizations were implemented and tested in the database to improve query performance. Each dataset ingested in the database has different spatial resolutions, and therefore needs to be resampled to the model resolution when generating its inputs. Since the model has a set of pre-defined spatial resolutions, caching can be employed to significantly reduce query times. Resampled data can be cached as new tables, as the result of a query precomputed from operating on the original raster table. Furthermore, each database table can be clustered, i.e. its rows reordered, based on the spatial proximity of each tile allowing for quicker access to time series of the same or nearby regions.

**Fig 3:**
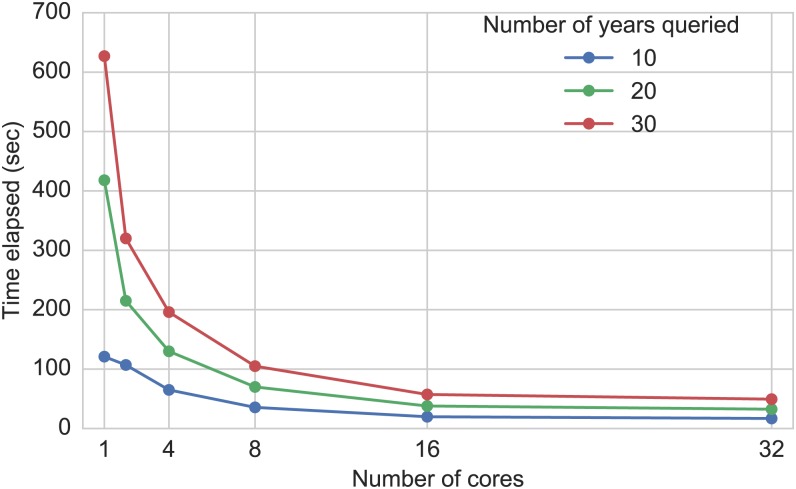
Database performance. Example database performance with multiple processors querying different number of years (10, 20, and 30).

Although PostgreSQL does not support multi-threaded queries explicitly, RHEAS utilizes database connection pools (i.e. a group of independent database connections) to execute queries in parallel. Fig 3 shows the performance of three queries with increasing number of processors used. Each query corresponds to a different period length retrieved from the database (10, 20, and 30 years) with the spatial domain being a basin of 3,013 pixels. The database performance actually scales very well as the number of processors increase from 1 to 16, with the scaling being almost linear. The single-core performance is actually 2 times slower than the dual-core query time due to the overhead introduced by the multi-threaded execution.

The dataset tables are grouped into schemas, with each schema representing a variable. For example, all precipitation-related datasets are contained within the precip schema. A list of the available datasets is shown in Table 2 along with the temporal and spatial resolution of each. Some of these data products have near-global coverage, while others have a regional focus (e.g. RFE2 over Africa or PRISM over the continental United States). RHEAS can automatically fetch each of these datasets and ingest them in the PostGIS database. A separate module was developed for each dataset, although function names for each module are identical (e.g. download) allowing for a common interface for the dataset functionality. Data providers use different standards to provide the datasets, both in terms of web services (e.g. OpenDAP, FTP) and file formats (e.g. NetCDF, HDF). Python decorators, a metaprogramming technique, can be used to transform the dataset module’s functions during runtime and facilitate code reuse. This allows defining a dataset module using only a Uniform Resource Locator (URL) address and specifications of the URL and file types. As an example, the CHIRPS rainfall dataset that is provided as a set of Geotiff files at a web repository is fetched by simply defining the function in Table 3 with 5 lines of Python code. The http decorator dynamically adds the functionality of retrieving the files defined with the url variable, while the geotiff decorator specifies how data can be extracted from the retrieved file.

**Table 2:** List of data products available in the RHEAS database.

Variable	Product	Spatial Resolution	Temporal Resolution	Period available	Reference
Precipitation	TRMM	0.25°	Daily	1998-Present	[23]
	GPM	0.1°	Daily	2014-Present	[24]
	PRISM	4 km	Daily	1981-Present	[25]
	CMORPH	8 km	Daily	1998-Present	[26]
	CHIRPS	5 km	Daily	1981-Present	[27]
	Princeton	0.25°	Daily	1948-Present	[28]
	RFE2	25 km	Daily	2000-Present	[29]
Temperature	NCEP	1.875°	Daily	1948-Present	[30]
	PRISM	4 km	Daily	1981-Present	[25]
	Princeton	0.25°	Daily	1948-Present	[28]
Wind speed	NCEP	1.875°	Daily	1948-Present	[30]
	Princeton	0.25°	Daily	1948-Present	[28]
Soil moisture	AMSR-E	25 km	Daily	2002-2011	[31]
	SMOS	40 km	Daily	2009-Present	[32]
	SMAP	36 km	Daily	2015-Present	[33]
Evapotranspiration	MOD16	1 km	8 days	2000-2013	[34]
Snow cover fraction	MODSCAG	500 m	Daily	2000-Present	[35]
	MOD10	500 m	Daily	2000-Present	[36]
Water storage	GRACE	300 km	Monthly	2002-Present	[37]
Leaf Area Index	MCD15	1 km	8 days	2002-Present	[38]
Meteorological forecasts	IRI	2.5°	Monthly	2000-Present	[39]
	NMME	0.5°	Daily	2000-Present	[40]

**Table 3:** Code snippet (URL shortened) defining the fetch function for the CHIRPS dataset module.

@geotiff
@http
def fetch(args):
url = “http://ftp.chg.ucsb.edu/…/chirps-v2.0.0:04d.1:02d.2:02d.tif.gz”
return url, args

The user can define which datasets should be ingested by creating a RHEAS configuration file that only optionally requires a bounding box, start and end dates along with the names of the datasets. If the optional arguments are not provided, RHEAS will query the database for the latest date that had been downloaded and update the dataset to today’s date. Table 4 shows an example configuration that can be used to download CHIRPS and NCEP data into the RHEAS database. The configuration file follows the INI format and is composed of sections and pairs of key/values. The domain section defines the geographical area, while each dataset has its own section and consequently its own parameters (e.g. period to download). Using this mechanism, RHEAS significantly simplifies retrieving of satellite and model datasets (including batch downloading) and automatically ingesting them in the PostGIS database for further processing by the user.

**Table 4:** Example configuration for downloading multiple datasets using RHEAS.

[domain]
minlat: -10
maxlat: 5
minlon: -20
maxlon: -12
[ncep]
[chirps]
startdate: 2000-1-1
enddate: 2000-2-1

### Hydrologic modeling

The hydrology model deployed in RHEAS is the Variable Infiltration Capacity (VIC) model [41]. VIC solves the energy and water balance over a gridded domain including a soil-vegetation-atmosphere scheme that models how moisture and energy fluxes between land and atmosphere are controlled by vegetation and soil. Numerous studies have utilized VIC to simulate the hydrology of large river basins continentally and globally (e.g. [42]), making it a good choice for the RHEAS software. The input requirements for VIC include meteorological data that force the model, and information on soil properties, elevation, and land cover. Although multiple sources exist for providing the information on land cover, soils etc., RHEAS has a set of datasets that are utilized to run VIC simulations at varying spatial resolutions (1°, 1/2°, 1/4° globally, and additionally 1/8° and 1/16° over the continental U.S.). Topography information used to partition each model grid cell into elevation zones is derived from the GTOPO30 global digital elevation model, which has a spatial resolution of 30 arc-seconds (∼1 km). Land cover information can be readily obtained from satellite datasets, such as the Moderate resolution Imaging Spectroradiometer (MODIS) global product that is generated at a 500-m spatial resolution [43]. Finally, VIC requires information on soil properties which are adapted from global and regional implementations of the VIC model [44, 45].

The VIC model is implemented as a class that contains the functionality of preparing the necessary input files (meteorological forcings, soil and land cover information), running the model executable, and ingesting the model output into the PostGIS database. The model class is encapsulated in the vic package, which also contains modules for saving/loading model state files (used during data assimilation) and parsing the user-provided configuration into model options.

### Data assimilation

Data assimilation allows for the optimal merging of model predictions and observations by statistically taking into account the errors in both. Earth Science observations can be ingested into RHEAS using a variety of data assimilation algorithms, with the default being the Ensemble Kalman Filter (EnKF). Additional assimilation algorithms included in RHEAS are the square root EnKF (SREnKF), and the Local Ensemble Transform Kalman Filter (LETKF). The EnKF [46] is a variant of the standard Kalman Filter optimal estimation algorithm and has been widely used in hydrology [13]. The SREnKF is similar to the EnKF, but avoids sampling errors introduced in the standard algorithm resulting in improved state estimates [47]. The LETKF is similar to the other ensemble filters, but performs the analysis (i.e. state update) independently for each model grid point, uses only observations that may affect specific grid point (i.e. localization), and offers algorithmic improvements that enhance the efficiency of the assimilation [48]. The assimilation algorithms within RHEAS have been implemented as abstract classes, i.e. can theoretically work with any model (assuming that they have the ability to restart simulations from a previously saved state), and utilize existing linear algebra libraries [49].

All data assimilation algorithms require an estimate of the model uncertainty, and in the case of the aforementioned techniques that uncertainty is captured by representing the hydrologic variable to be estimated stochastically with an ensemble. Ensembles of model simulations can be generated either by perturbing model forcings and/or parameters (e.g. [19]), or sampling appropriately from climatology. The generation of the ensemble can be controlled by the user (via the configuration file), with all the aforementioned options implemented within RHEAS. When an observation becomes available, the model state is updated leading to an optimal estimate (in terms of least squares). When RHEAS is in nowcast mode the simulation proceeds until the next observations become available, whereas in forecast mode observations are assimilated up to the forecast initialization date after which the model(s) run “free”.

**Fig 4:**
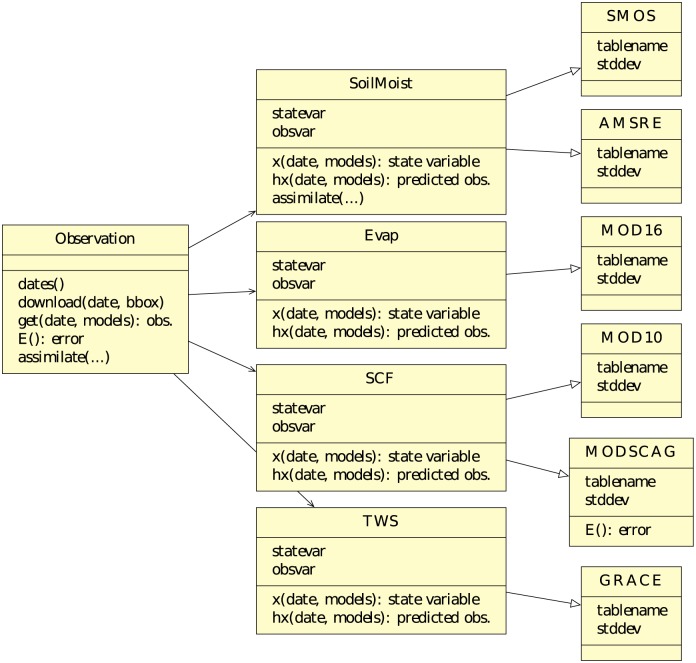
UML observation-class diagram. UML diagram of classes representing observational datasets that are assimilated (the SMAP class is omitted for visualization purposes).

In order to streamline the assimilation of multi-sensor observations, RHEAS is using an object-oriented software design maximimizing code reuse [50]. Fig 4 shows a UML diagram of the software classes that represent the observational datasets and their inter-relationships. An abstract class type (Observation) contains the functionality to download the different data products and query the database for the latest data available, retrieve the observation vector for a specific date, generate the observation errors, and perform the data assimilation. The Observation abstract data type is implemented as a set of parent classes for each observation variable and encapsulates parameters and functions specific to that variable. For example, the soil moisture class (SoilMoist in Fig 4) defines the state (total-column soil moisture) and the observed variable (top-layer soil moisture), and functions for estimating the predicted measurements and deriving the state ensemble. Additionally, if observation-specific methods for data assimilation exist, such as bias correction for soil moisture [51], they are added to the level of this abstract class and are transparently available to each observation sub-class. The latter sub-classes encapsulate parameters specific to a data product (table name in the database, a standard deviation for its error) and inherit their functionality from the parent abstract classes although these can be overriden. For example, a different function that generates the observation error can be defined for the MODSCAG class (Fig 4). Alternatively, uncertainty in the observations can be defined by the user in the RHEAS configuration file (stored locally on the user’s computer) by providing the name of a propability distribution (PDF) and a set of parameters. Most current approaches to specifying uncertain parameters in statistical software use markup language representations, either through structured formats such as XML (EXtensible Markup Language) and JSON (JavaScript Object Notation) or an actual Application Programming Interface (API), with a prominent example being UncertML [52]. A slightly different approach was taken in RHEAS, where dynamic module loading from the Scipy library [53] was used to provide the function that samples the PDF to generate the observation errors. Scipy provides a large number of probability distributions, with RHEAS having fallback functions in cases where the user either defines an unavailable distribution or does not provide enough parameters for it. Furthermore, additional assimilation algorithms such as the Particle Filter [54] can be implemented within RHEAS, by utilizing the Observation classes and the EnKF class as a template.

### Model coupling

The modular architecture of RHEAS and the ability to access data in a model-agnostic manner (via the PostGIS database) allow the one-way (i.e. offline) coupling with other environmental models by simply developing an interface against the database itself rather than the hydrologic model. As an example, an agricultural model has been coupled within the RHEAS framework enhancing the software’s applicability. The crop model included in RHEAS is based on the Decision Support System for Agro-technology Transfer (DSSAT) modeling system [55]. DSSAT is a process-based model that simulates the growth, development and yield of a crop under given management practices and soil properties (e.g. fertility, water holding capacity). Additional input to DSSAT includes time series of weather variables (rainfall, air temperature, and net solar radiation) that are used to drive the soil hydrology physical model component within DSSAT. The latter interacts with the crop model component of DSSAT simulating the plant’s phenology, morphology, and yield.

The DSSAT model implementation used within RHEAS is a modified version of the baseline model that can stop and restart at arbitrary times, whereas crop models generally run continuously from sowing until maturity, failure or harvest by design [56]. This modification was necessary (not just for DSSAT but any model that is coupled within RHEAS) in order to facilitate data assimilation of soil moisture and LAI observations during different phases of crop growth. Moreover, it has been adapted to be deployable over a gridded domain in contrast to the original DSSAT version that is point-based.

### Simulation modes and configuration

Each RHEAS simulation begins with the parsing of a user-provided configuration file that is populated with various simulation parameters. The configuration file follows the INI format, with each section corresponding to the type of simulation (nowcast or forecast) and the model used (VIC and/or DSSAT). At a minimum the simulation configuration requires the type of model to be used, the period of simulation, a vector GIS file that defines the model domain, the spatial resolution, and a name for the simulation. Similarly, the VIC model configuration requires the source of meteorological data, and a set of output variables that are written to the RHEAS database. Additional options can be set by the user through the configuration file, although defaults have been preset in order to simplify the deployment of model simulations for non-expert users.

**Fig 5:**
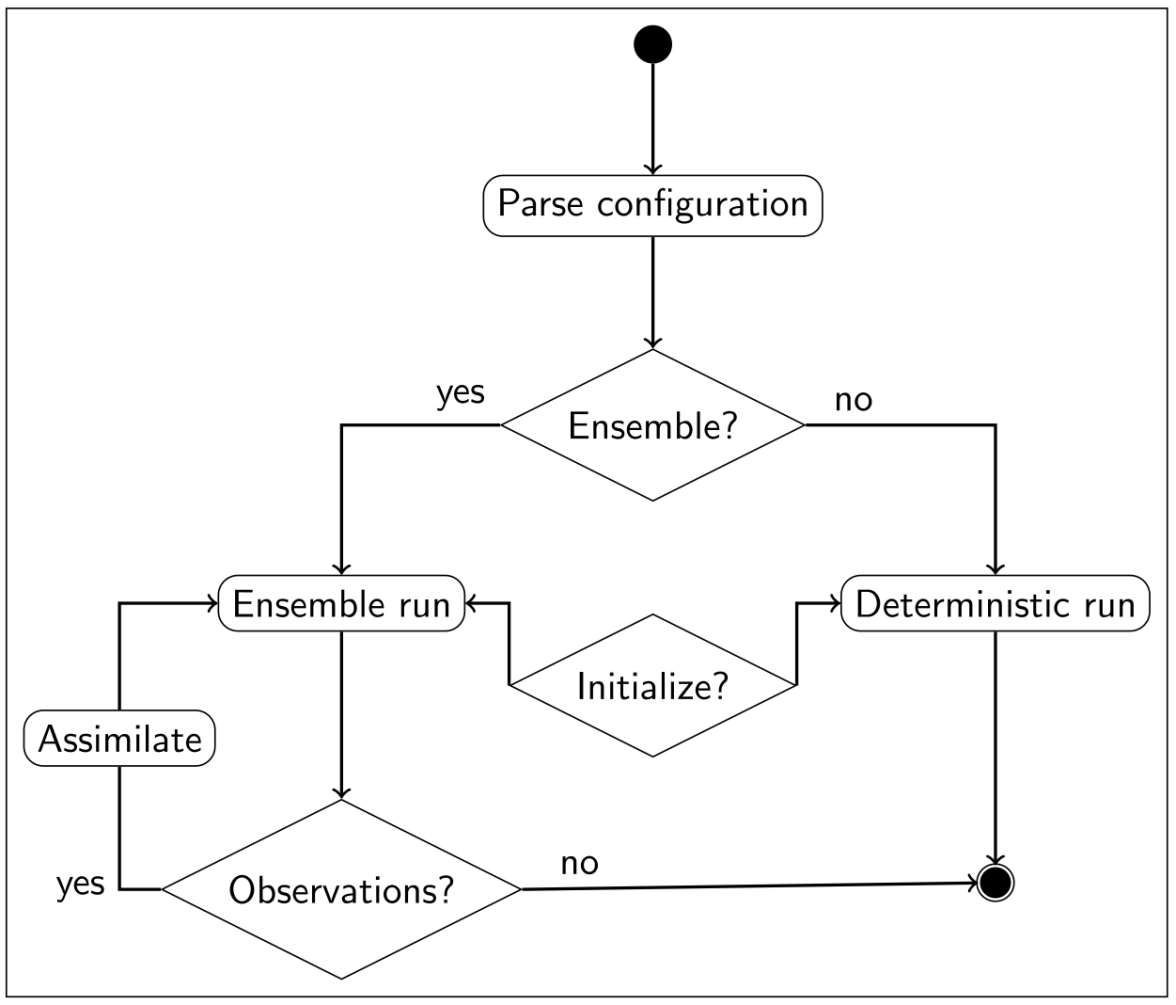
Nowcast diagram. Sequence diagram for the nowcasting mode of RHEAS.

Nowcast simulations (Fig 5) can either be performed deterministically (i.e. single model realization) or stochastically (i.e. ensemble of models). Depending on user input the models can be initialized from a saved state to ensure proper spin-up, which can itself consist of multi-year simulations. If observations are available during the simulation period, they can be assimilated into the model sequentially. The update frequency can be set by the user, since RHEAS supports keywords such as “weekly” and “monthly”. Moreover, data assimilation can only be performed with a stochastic simulation since the assimilation algorithms implemented within RHEAS require an ensemble to describe the model uncertainty.

**Fig 6:**
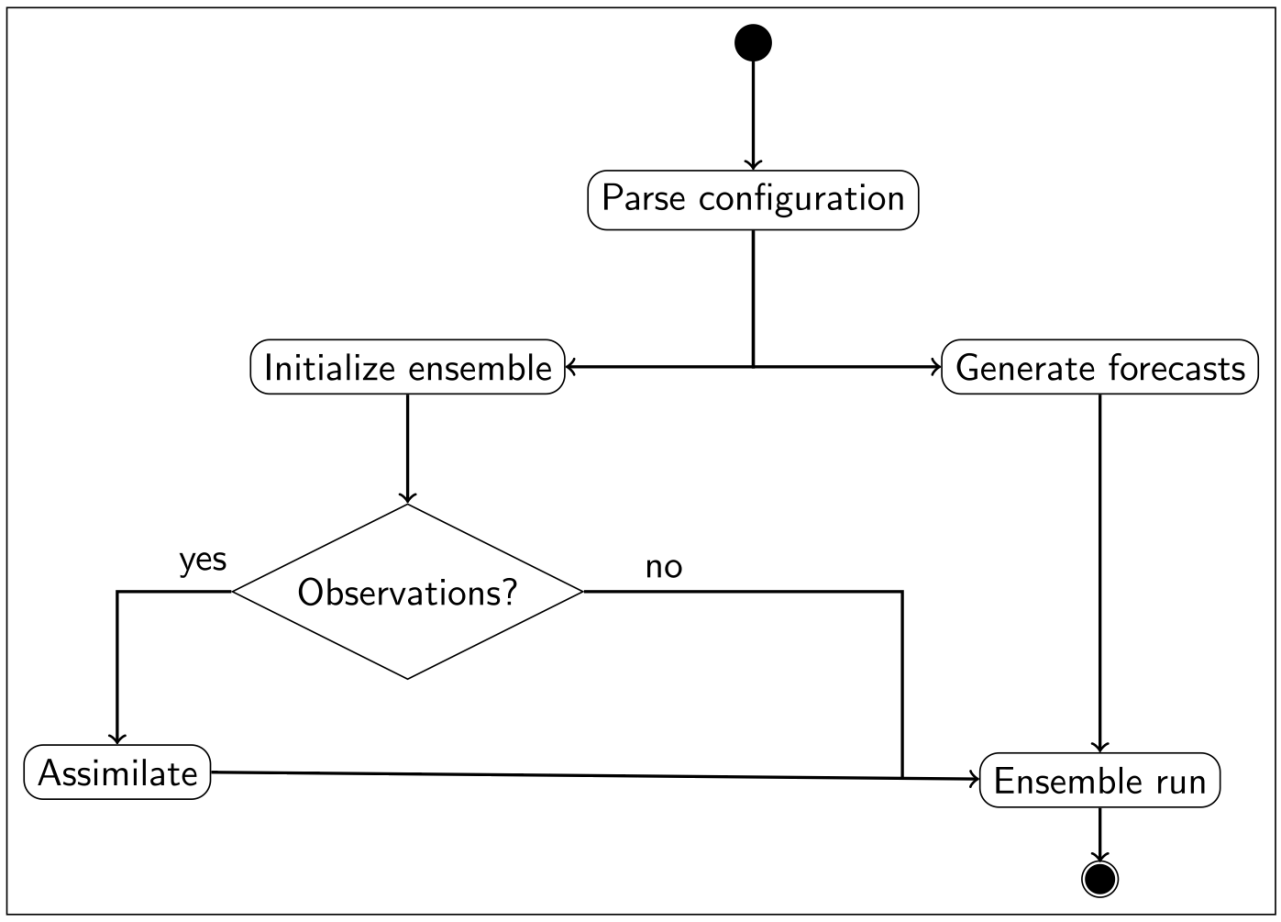
Forecast diagram. Sequence diagram for the forecasting mode of RHEAS.

Similar to the nowcasting mode, forecast simulations (Fig 6) commence with parsing the user-provided configuration file. The hydrologic model requires meteorological forecast data that need to be disaggregated spatially and/or temporally to match the model’s spatial and temporal resolution. The forecast methods implemented in RHEAS are probabilistic, with an ensemble of models spun up to the forecast initialization date. If observations are available on that date, they can be assimilated into the model and the forecast simulation is then launched. The simulation period depends on the duration of the meteorological forecast (e.g. 3 months for seasonal forecasts), with the entire model ensemble being saved into the database at the end. The meteorological forecasts can be generated either by resampling from climatology or from an atmospheric model.

Whenever a stochastic simulation is performed, an ensemble of model output is saved in the RHEAS database. Ensemble simulations are performed with multi-threaded processing, i.e. each ensemble member is run by a different CPU core, hence accelerating the simulation time. A byproduct of ensemble simulations is the derivation of an uncertainty estimate for each output variable, which can be expressed as the ensemble’s standard deviation. Although more sophisticated techniques of estimating uncertainty exist such as Bayesian (excluding Kalman Filters) methods [57], their implementation were beyond the scope of the initial release of RHEAS but could be added potentially to enhance the prediction system. Based on basic decision theory, uncertainty can be considered as a representation of a set of possible states or outcomes with a known probability of occurrence [58]. A decision-maker can choose from a set of possible alternative actions that correspond to each outcome, making even the simple uncertainty estimate from RHEAS potentially useful.

Table 5 shows an example configuration file for a nowcast simulation that assimilates SMOS soil moisture and GRACE water storage observations. The output variables, soil moisture and evaporation, will be written in the database under the schema testing as raster tables soil_moist and evap respectively with each row corresponding to each model time step (daily in this case). Since an ensemble simulation is performed using this configuration, a column specifying the ensemble member number will be added to the aforementioned output tables while additional tables containing rasters of the standard deviation for each variable will be added.

**Table 5:** Example RHEAS configuration file for a nowcast simulation.

[nowcast]
name: testing
startdate: 2012-3-1
enddate: 2012-9-30
basin: domain.shp
resolution: 12km
ensemble size: 10
[vic]
precip: prism
temperature: prism
wind: ncep
save: soil_moist, evap
observations: smos, grace

## Application examples

Here, the RHEAS framework was implemented in three case study areas in order to demonstrate and evaluate its capabilities before potentially being deployed operationally, with example results described below. Table 6 shows the execution times for each of the case-study simulations.

**Table 6:** Execution times and domain size for each of the case-study simulations (using a 3-GHz 8-core Intel Xeon E5 processor).

Case study	Domain size (pixels)	Execution time (hrs)
California drought nowcasting	753	0.84
Colorado flow forecasting	3,402	3.37
Kenya crop yield nowcasting	761	1.37

### Drought nowcasting in California

**Fig 7:**
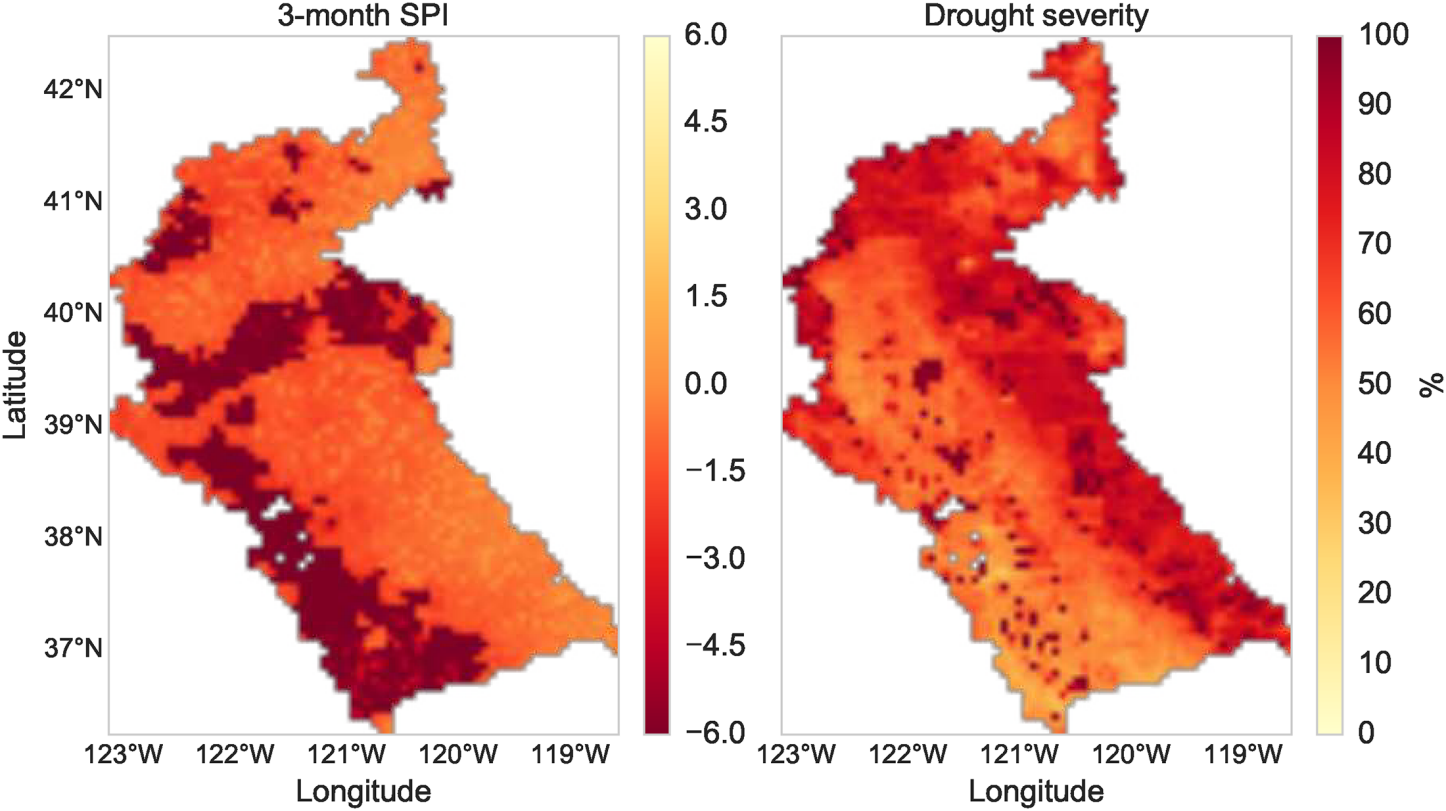
Drought data products. Maps of the 3-month Standardized Precipitation Index (left) and agricultural drought severity (right) on 1 July 2014 over the Sacramento/San Joaquin basin.

The Sacramento and San Joaquin river basins are located in California and cover most of the Central Valley, which is one of the most productive agricultural areas in the United States. Water resources in the region have been adversely affected by a severe drought that began in the winter of 2011 [59]. Consequently, accurate information on drought characteristics such as the ones produced by RHEAS become very important for water resources managers and practitioners. Fig 7 shows two example maps of drought indicators over the basin on July 2014. The left map shows the 3-month Standardized Precipitation Index (SPI), which is based on the probability of seasonal precipitation and reflects short-term moisture conditions [60]. Agricultural drought severity (Fig 7, right) is derived from the root zone soil moisture expressed as a percentile of the 1981-2010 climatology using the methodology of [61].

**Fig 8:**
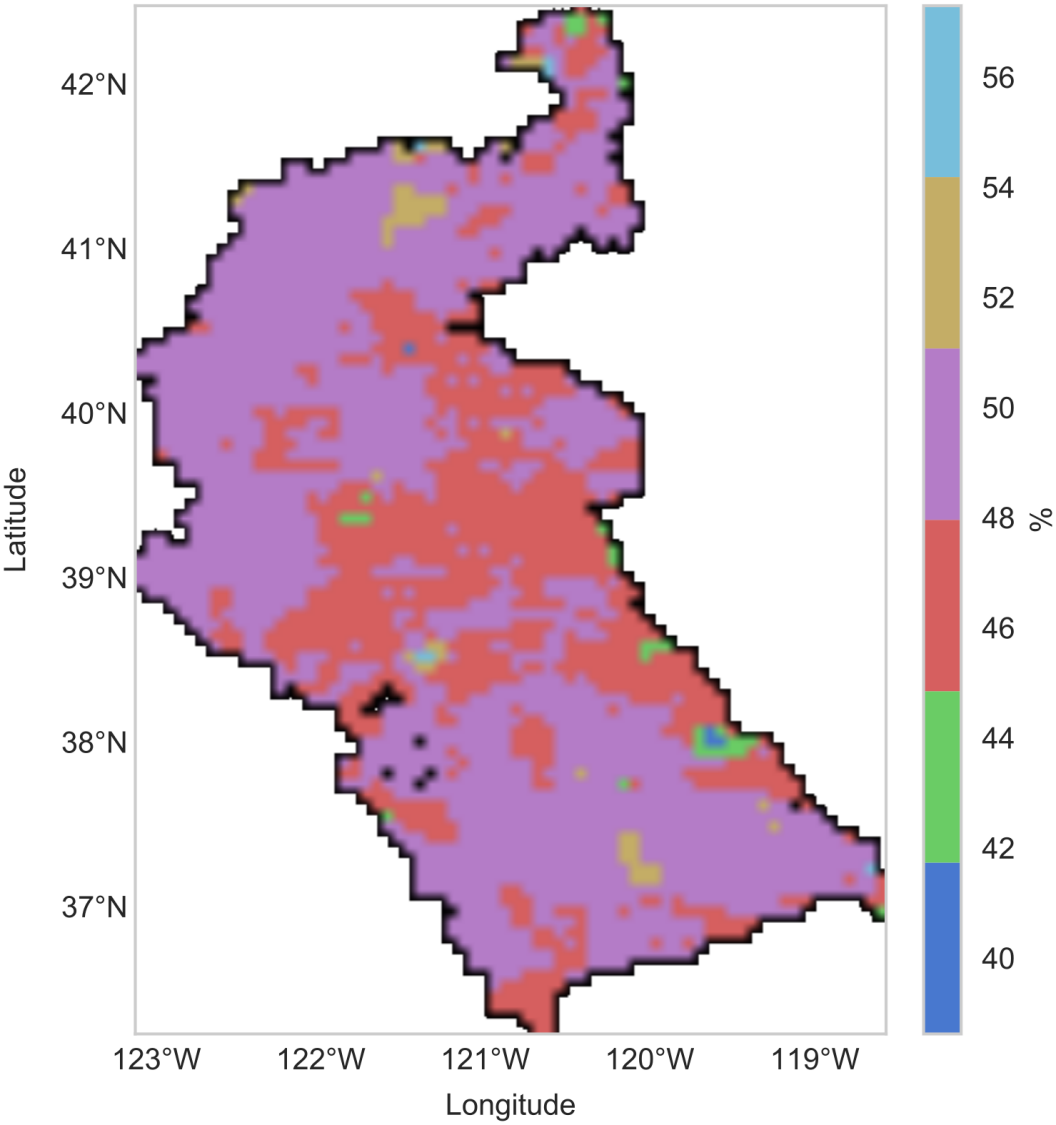
Uncertainty map. Map of uncertainty (derived from ensemble 1*σ*) in soil moisture over Sacramento/San Joaquin river basin on 31 August 2014.

The simulations performed to produce these maps used the PRISM dataset to derive precipitation and air temperature forcings for the VIC model, while NLDAS was used to derive wind speed. In addition to the drought data products generated from RHEAS, uncertainty estimates for all simulated hydrologic variables were available. Fig 8 shows a map of uncertainty (expressed as the percentage standard deviation of a 5-member ensemble) in soil moisture over the basin at the end of August 2014.

### Flow forecasting in the Upper Colorado River

**Fig 9:**
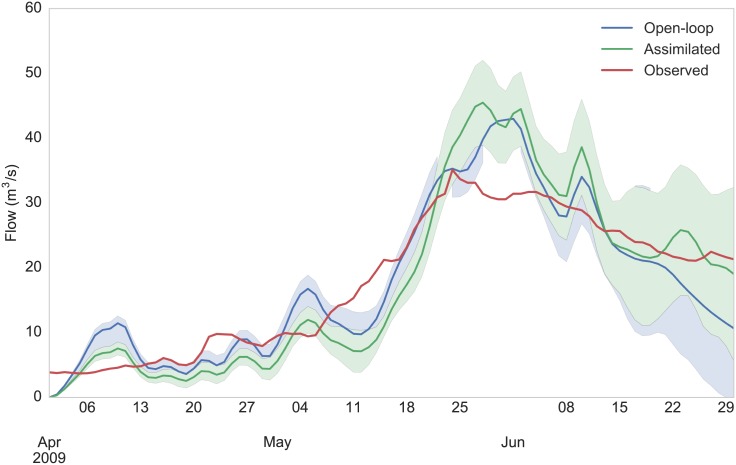
Streamflow forecast plot. Time series of forecasted (open-loop and assimilated) and observed streamflow at Taylor River with forecasts initialized on 1 April 2009. Forecasts are bounded by the 25th and 75th percentile of the ensemble.

The Upper Colorado River plays a very important role for the water resources of the western United States [62], having a rather diverse intra-basin physiography (e.g. elevations range between about 1,000 to more than 4,000 m). Snow controls the timing and magnitude of peak runoff in the basin [63], and therefore has significant implications for water supply management. Observations of snow cover can potentially improve the estimation of streamflow and its forecast skill. In order to test that hypothesis, seasonal forecasts of streamflow were generated using RHEAS at the Colorado River Basin Forecast Center’s forecast points. MODIS snow cover observations were assimilated during forecast initialization (1 April 2009), and ESP was used to simulate hindcasts of streamflow. Streamflow was generated by using the offline VIC river routing model [64] with inputs from the RHEAS simulations. Fig 9 shows time series of streamflow forecasts along with the actually observed streamflow at Taylor River near Altmont, Colorado (ALTC2 station). In contrast to open-loop forecasts, assimilated forecasts ingest MODSCAG observations during the initialization date. The forecast ensemble means show that the assimilation of the snow cover observations improved the streamflow forecast skill after mid-May. The improved snow water equivalent (SWE) estimation during forecast initialization manifests as improvements in streamflow when snowmelt begins to have a significant contribution to the basin’s outflow. Additionally, the ensemble spread from both simulations is shown in Fig 9, in the form of the 25th and 75 percentile bounds, with the assimilated forecast range being smaller than the open-loop one suggesting a reduction in uncertainty.

### Crop growth nowcasting in Kenya

**Fig 10:**
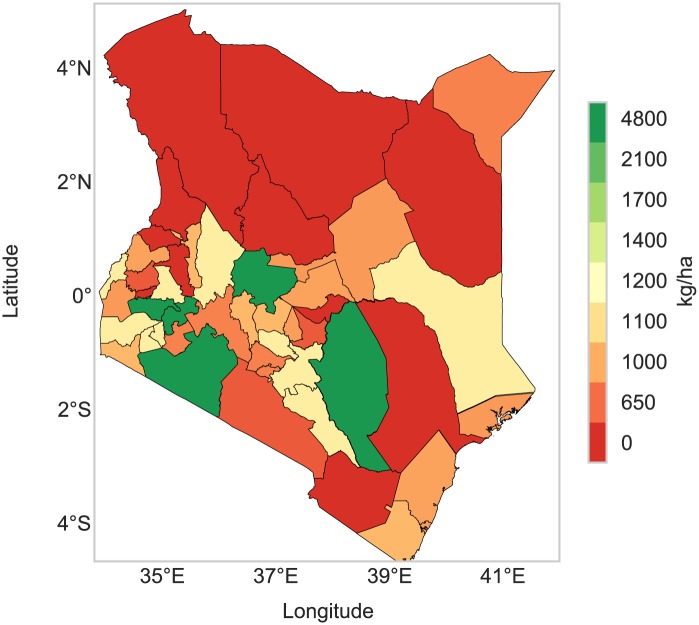
Crop yield map. Map of maize yield over Kenya in 2011 (first planting season) dissagregated to county level.

RHEAS has been implemented over several countries in the East Africa region, with the goal of producing hydro-agricultural nowcasts and forecasts that can eventually be used to inform decision-making by farmers. Fig 10 shows an example map of simulated maize yield after the earlier planting season in 2011 over Kenya. The yield estimates have been spatially aggregated at the county level, although the finest scale of the simulated output can be defined by a user-provided GIS vector file. The DSSAT model was driven by soil moisture, net solar radiation that were derived from the coupled VIC simulation, as well as LAI, rainfall and air temperature derived from MODIS observations and the CHIRPS and NCEP datasets respectively. A first-order estimate of planting dates were obtained from a global, 1/2° resolution crop calendar dataset [65]. These estimates have been ingested in the RHEAS database and were used for this simulation, while maize cultivar information (3 varieties) were taken from the Agricultural Model Intercomparison Project [66]. No irrigation and low fertilization were assumed for this simulation, since most of Kenya’s agriculture is rain-fed [67] while getting specific information on fertilizer application can be difficult. A drought occurred in East Africa during 2011 affecting agricultural productivity, with counties in Kenya having low yield (Fig 10). Although county-specific yield data were not available, a crude comparison with the national scale with FAOSTAT reported a yield of 1,584 kg/ha for maize while RHEAS simulated a yield of 1,364 kg/ha.

**Fig 11:**
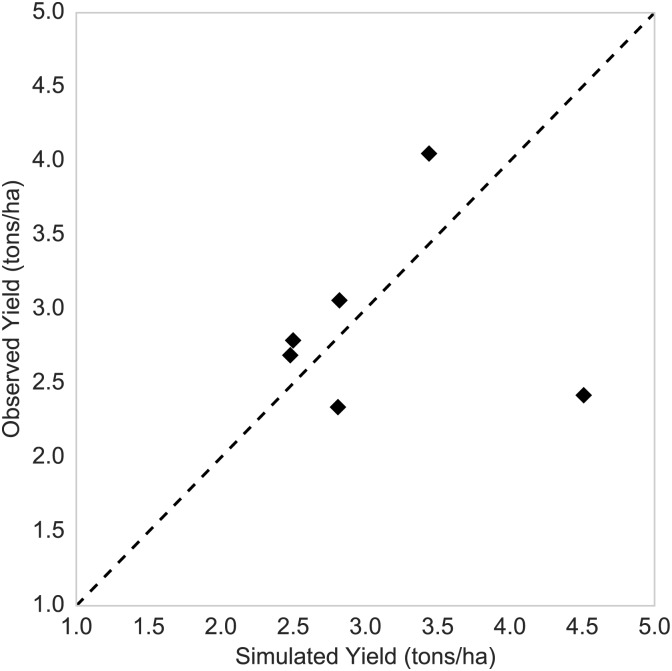
Crop yield validation. Comparison of simulated and observed maize yields over the Nzoia River basin during the earlier growing season of 2000-2006.

Detailed validation data are difficult to obtain, but yield observations for maize were available over the Nzoia River basin during 2000-2006. The basin has an area of 17,392 km^2^, and a RHEAS simulation at a 25km resolution produced yield estimates during the earlier growing season of the same period as the observations. Fig 11 shows the comparison of the simulated and the observed yields, and with the exception of 2003 when RHEAS significantly overestimated yield (4.51 tons/ha versus 2.42 tons/ha), the model shows reasonably good agreement.

## Summary and future directions

A software framework, RHEAS, that facilitates the deployment of water resources simulations through the ingestion and assimilation of a variety of datasets (including satellite observations) was presented along with three case studies. RHEAS has a spatially-enabled PostgreSQL/PostGIS database at the core with various datasets, both satellite and model-based, being ingested automatically. Apart from the study area, simulation period, and names of input and assimilated datasets, the user does not need to specify any additional parameters, although a programming API (in Python) allows the further customization of the system. Nowcast and forecast simulations can be performed with or without assimilating satellite observations, with the latter representing most of the water cycle components. The RHEAS database allows for the different modeling components to interface against it, simplifying the coupling of an crop growth model. Moreover, the GIS features of the database facilitate the dissemination of the RHEAS data products to diverse platforms (desktop, web and mobile). The output data products include an exhaustive set of hydrologic variables, with each having an uncertainty estimated associated with it.

Compared to similar modeling systems that either require extensive configuration or scripting a solution custom to a specific end-user, RHEAS allows the implementation of a nowcast and/or forecasting system with minimal inputs from user automating and abstracting many of the details away. Although the case studies presented did not include extensive validation, they showcased the ability of RHEAS to generate a suite of data products in relatively diverse contexts. The spatial scales that RHEAS is applicable are governed by the hydrology model at its core, VIC, which has been implemented at resolutions ranging from 1/16° to 1°. Therefore the minimum spatial scales for the current version of RHEAS should be on the order of 5 km. Nonetheless, specific regional applications may require further calibration (currently achievable with external software tools) of the VIC model parameters and validation against either in-situ or satellite measurements.

The modular architecture and design of RHEAS could allow for potential modifications in the future that could enhance its applicability. In the context of decision-making, examples could include the use of a soil moisture change product to provide outlook on the crop growth potential, or the drought onset/duration product to plan for food storage. Apart from coupling other types of models (currently the crop growth model DSSAT is available), such as a hydrodynamic model making the system suitable for flood forecasting applications at even sub-hourly time steps. In addition, other hydrologic models can be added within RHEAS to supplement VIC creating a multi-model ensemble, which could improve predictability. Extension of the RHEAS software framework with new models will require the development of modules that implement I/O between the model and the PostGIS database, and execution of the model physics (including the preparation of its custom input files). Currently there are plans to couple additional agricultural models into the framework representing different crops (e.g rice, cacao) but we anticipate that code contributions from the open source community will further enhance the framework’s applicability.
